# Causal association of genetically determined plasma metabolites with osteoarthritis: a two-sample Mendelian randomization study

**DOI:** 10.3389/fmed.2024.1396746

**Published:** 2024-06-28

**Authors:** Qingsong Fu, Xinhua Yuan, Weibin Wang, Xinyou Han, Jiakai Zhang, Junlong Wu, Yao Wang

**Affiliations:** Department of Trauma and Orthopaedic, Ningbo No. 2 Hospital, Ningbo, Zhejiang, China

**Keywords:** metabolites, osteoarthritis, Mendelian randomization, causal, two-sample

## Abstract

**Background:**

We aimed to elucidate the causal relationship between plasma metabolites and the vulnerability to Osteoarthritis (OA), encompassing both hip OA and knee OA.

**Methods:**

We conducted a two-way two-sample Mendelian randomization (MR) analysis to investigate the association of 1,400 plasma metabolites with OA. The Inverse Variance Weighted (IVW) model served as the primary two-sample MR Analysis method, with supplementary analysis using the Weighted Median (WM) and MR Egger methods. To ensure the robustness of our findings, sensitivity analyses were performed, incorporating Cochran’s Q test, MR-Egger intercept test, MR-PRESSO, and Leave-One-Out analyses. To validate the identified metabolites, we utilized the Steiger test and linkage disequilibrium score regression.

**Results:**

A total of 94 plasma metabolites were associated with osteoarthritis, with 60 associated with hip OA and 106 associated with knee OA. IVW analysis revealed that tryptophan levels showed the strongest positive association with hip OA (OR [95% CI]: 1.119 [1.024, 1.223]), while X-24757 levels exhibited the highest positive association with knee osteoarthritis (OR [95% CI]: 1.095 [1.032, 1.162]). Ethylparaben sulfate levels were found to have the greatest positive association with hip OA (OR [95% CI]: 1.118 [1.015, 1.231]). Notably, the plasma metabolite X-2475 showed a strong robust random effect across all three types of osteoarthritis. Metabolic pathway analysis revealed that the pathogenesis of osteoarthritis in the hip was mediated by acetylarginine, specifically in four important metabolic pathways: ethanol degradation (*p* = 0.044), amino sugar metabolism (*p* = 0.090), fatty acid biosynthesis (*p* = 0.095), and aspartate metabolism (*p* = 0.097816).

**Conclusion:**

There is a significant association between tryptophan levels and the risk of hip OA, as well as X-24757 levels and the risk of knee osteoarthritis. Additionally, X-24757 levels are also linked to the risk of hip OA. Moreover, this study has identified four crucial metabolic pathways in hip osteoarthritis, which are all regulated by acetylarginine. These findings provide valuable insights into potential biomarkers for OA and highlight potential pathways for its prevention and clinical intervention.

## 1 Introduction

Osteoarthritis (OA) is a prevalent degenerative disease that affects joint-related tissues, such as articular cartilage, leading to joint instability and reduced quality of life (1, 2). In 2020, more than 500 million people globally, accounting for 7% of the population, experienced OA (3). Underlying factors such as trauma, obesity, and congenital abnormalities contribute to weakened cartilage and chronic OA (4). Nevertheless, the pathogenesis of OA remains unclear, resulting in a lack of effective preventive measures for this chronic condition.

Recent evidence suggests a potential connection between plasma metabolites and OA, indicating that metabolic disorders may play a significant role in OA development (5–9). The influence of plasma metabolites on OA progression has been reported in previous studies (10, 11). However, these investigations primarily focus on the mechanism of OA without providing clear conclusions. Traditional observational epidemiological studies are constrained by small sample sizes and potential biases from confounding factors, including reverse causality. Randomized controlled trials, although the gold standard for establishing causality, are complicated and resource-intensive, making them challenging to conduct. Furthermore, their strict inclusion and exclusion criteria may limit the generalizability of findings. Therefore, a comprehensive and thorough analysis is urgently needed to reveal the causal role of metabolites in the mechanism of OA.

Genetic approach represents a valid method to assess causality free from confounding or reverse causality bias (12). To overcome limitations as mentioned above, Mendelian randomization (MR) can be employed. As a form of “randomized controlled trial” in nature, MR uses genetic variants as IVs to infer potential causal relationships between genetically influenced exposures and outcomes, providing unbiased estimates (13). Egger Mendelian randomization (MR-Egger) and weighted median MR provide statistical tests for presence of pleiotropic effects of the single-nucleotide polymorphisms (SNPs) under analysis (14, 15).

Therefore, this study utilized MR to assess the potential causal effect of plasma metabolites on OA, considering plasma metabolites as the exposure and OA as the outcome, encompassing both hip OA and knee OA. Identification of plasma metabolites with potential OA risks helps our health policies.

## 2 Methods

### 2.1 GWAS data of human plasma metabolites

Our investigation utilized summary statistics from extensive Genome-Wide Association Studies (GWASs) conducted on 1,091 plasma metabolites and 309 metabolite ratios, which encompassed a cohort of 8,299 individuals of European ancestry from the Canadian Longitudinal Study on Aging (CLSA). Out of the 1,091 plasma metabolites analyzed, 850 were identified with known characteristics that covered eight super pathways, including lipid, amino acid, xenobiotics, nucleotide, cofactor and vitamins, carbohydrate, peptide, and energy pathways. The remaining 241 metabolites were classified as either unknown or “partially” characterized molecules. These metabolites which were unknown or “partially” characterized were listed in their codes generally. The code of this kind of metabolites will be listed in this study. Furthermore, GWAS summary statistics for 1,400 plasma metabolites can be accessed through the GWAS Catalog platform.^1^ Ethical approval was obtained from the FinnGen steering committee for all selected GWASs within the FinnGen Consortium, and individuals provided written informed consent.

### 2.2 GWAS data for OA

GWASsummary statistics for osteoarthritis (ukb-b-14486), hip OA (ebi-a-GCST007091), and knee osteoarthritis (ebi-a-GCST007090) were retrieved from the IEU Open GWAS Project.^2^ The GWAS summary statistics for osteoarthritis include 38,472 cases and 424,461 controls, with information on 9,851,867 single nucleotide polymorphisms (SNPs). On the other hand, the GWAS summary statistics for knee osteoarthritis comprise 24,955 cases and 378,169 controls, and data on 29,999,696 SNPs. Lastly, the GWAS summary statistics for hip osteoarthritis consist of 15,704 cases and 378,169 controls, encompassing 29,771,219 SNPs. OA refers to general osteoarthritis, which includes various joint locations such as the hands, spine, and other locations. This general category of OA also includes hip and knee OA but does not exclusively focus on these two joints. Specific categories of “hip OA” and “knee OA” refer exclusively to osteoarthritis in the hip and knee joints, respectively. Notably, all data were sourced from online databases, and participants provided written informed consent for their involvement in the study.

### 2.3 Selection of instrumental variables (IV)

The selection of instrumental variables (IVs) in this MR analysis is grounded on three fundamental assumptions (16). The three fundamental assumptions including: (1) they must have a strong correlation with exposure factors; (2) there should be no correlation between instrumental variables and confounding factors; and (3) there should be no direct correlation between instrumental variables and outcome events, with the relationship only occurring through exposure factors. Supplementary Figure S1 illustrates these relationships, with solid lines indicating associations and dashed lines indicating the absence of association. Initially, SNPs associated with each metabolite at a significance level of *p* < 1 × 10^−5^ were extracted. Subsequently, the clumping functionality within the R software was utilized to ascertain independent variants, whereby a genomic distance of 500 kilobases (kb) was applied using the European 1,000 Genomes Project Phase 3 reference panel, with the linkage disequilibrium threshold set at R^2^ < 0.001 (17). Lastly, to assess the robustness of the chosen SNP as a valid IV, the F-statistic was computed for each metabolite. Typically, a threshold of *F* > 10 is recommended for advancing with further MR analysis.

### 2.4 Metabolic pathway analysis

Metabolic pathways were analyzed using the web-based tool Metaconflict 4.0.^3^ The functional analysis and pathway enrichment analyses module was employed to identify potential metabolites that may be associated with the biological processes or pathways involved in OA. The Small Molecule Pathway Database (SMPDB) and the Kyoto Encyclopedia of Genes and Genomes (KEGG) database were utilized in this study. A significance level of 0.10 was set for the pathway analysis.

### 2.5 Statistical methods

Utilizing inverse variance weighting (IVW) as the primary method for causal analysis, with MR Egger and weighted median as supplementary methods to validate the results further; When meeting all three assumptions of Mendelian randomization, IVW is used to combine multiple SNPs for MR analysis. The IVW method assumes that each SNP tool provides the same effect estimate and that no SNP exhibits horizontal pleiotropy. However, this ideal model may not hold in reality, as instrumental variables can have pleiotropic effects on both exposure and outcomes. The other methods serve as complements or enhancements to IVW.

For instance, the weighted median method in causal estimation allows more influential genetic variations to have a greater impact on the model, with increased weight. This method can offer unbiased estimates even if a significant portion of instrumental variables are invalid. The MR-Egger intercept test was utilized to detect horizontal pleiotropy, while MR-PRESSO was used to identify outlier SNPs. In cases where the exclusivity hypothesis is not confirmed, such as when conducting MR Egger regression to assess for pleiotropy, a *p*-value of less than 0.05 suggests the presence of horizontal pleiotropy. Additionally, when utilizing Cochran’s Q-test to evaluate the heterogeneity of instrumental variables, a p-value greater than 0.05 indicates a low probability of heterogeneity. In MR Egger regression, a *p* value greater than 0.05 for the intercept indicates that gene pleiotropy may not significantly impact the causal analysis results. MR Egger regression can relax the assumption of no horizontal pleiotropy between SNPs and provide accurate causal effect estimates even if all instrumental variables are flawed. Any identified outlier SNPs were excluded from the MR Analysis to ensure result reliability and consistency. The inverse variance weighted results were analyzed using the ‘leave one out’ method to assess the robustness of the findings. After removing each SNP individually, all results remained significant at *p* < 0.05. This consistency with the inverse variance weighting method in causal effect analysis suggests that no single non-specific SNP significantly influences the causal estimation results.

Additionally, a complementary sensitivity analysis was carried out using weighted pattern analysis. In cases where the inverse-variance-weighted analysis yielded statistically significant results (*p* < 0.05) without evidence of horizontal pleiotropy or heterogeneity, the results were considered positive if the direction of β values remained consistent, even if other methods did not yield significant results. If horizontal pleiotropy was present but no heterogeneity was detected, the MR-Egger method was used. Conversely, in cases of observed heterogeneity but no pleiotropy, the analysis was conducted using either the weighted median method or the random-effects IVW method. To address potential reverse causation between outcome and exposure variables, the Steiger causality test was employed to determine the correct direction of causality. The analysis was carried out using R 4.2.2 software and the R packages ‘TwosampleMR’ and ‘MR-PRESSO’.

## 3 Results

**Figure 1:**
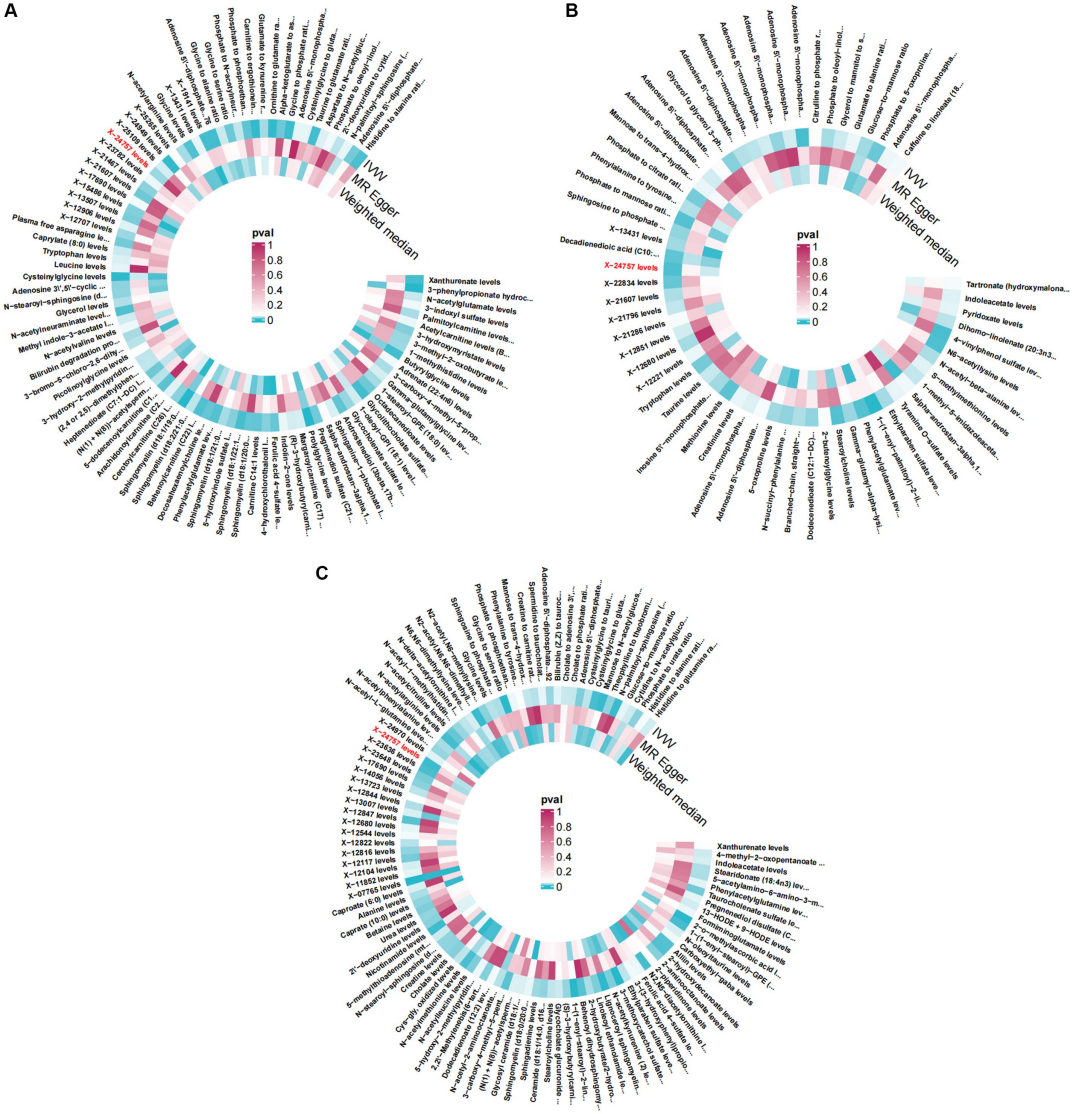
Circular Heatmap. Causitive relationship between Plasma Metabolites (*P_IVW_* < 0.05) and osteoarthritis, osteoarthritis (hip) and knee osteoarthritis using different MR methods. OR, odd ratio. **(A)** Causitive relationship between plasma Metabolites and osteoarthritis **(B)** Causitive relationship between plasma Metabolites and osteoarthritis (hip) **(C)** Causitive relationship between plasma Metabolites and knee osteoarthritis.

**Figure 2:**
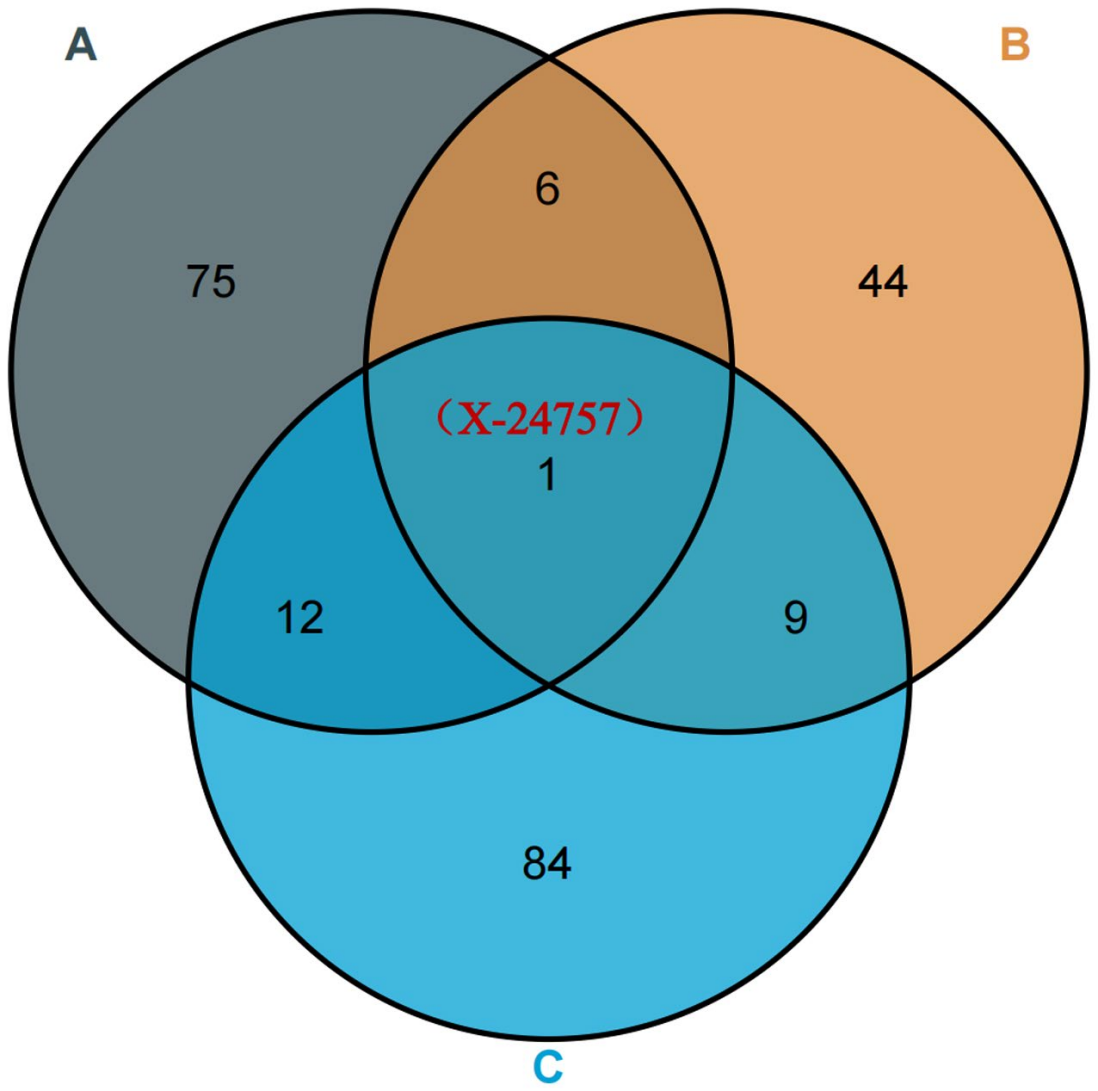
Forest plot for causal relationships of robust overlapped plasma metabolites on osteoarthritis and osteoarthritis (hip) utilizing Mendelian randomization. The change in the odds ratio (OR) of osteoarthritis, osteoarthritis (hip) per one-SD rise in the plasma metabolite level or metabolite ratio is shown by OR and 95% confidence interval.

The number of individuals at each stage and the phenotypic exposures and outcomes can been seen at Supplementary Figure S2. After conducting a series of quality control procedures, it was observed that 94 plasma metabolites were correlated with Osteoarthritis, while 60 plasma metabolites showed an association with hip OA, and 106 were linked to knee Osteoarthritis (Supplementary Table S7). A total of 96 SNPs were identified in relation to Osteoarthritis, and 99 SNPs were associated with hip OA (refer to Table 1; Supplementary Tables S1, S2). Notably, SNPs with *F* values greater than 10 were prioritized. Furthermore, utilizing IVW analysis, evidence indicated that 7 specific plasma metabolites were associated with both Osteoarthritis and hip OA (Figures 1, 2; Supplementary Tables S4, S5, S7).

**Table 1:** Causal effects of significant plasma metabolites (*P_IVW_* < 0.05) on osteoarthritis and osteoarthritis (hip).

Exposure	Outcome	Method	nsnp	*p* value	OR (95%CI)
Phenylacetylglutamate levels	Osteoarthritis	Inverse variance weighted	13	0.006	0.995 (0.991, 0.999)
		Weighted median	13	0.174	0.996 (0.991, 1.002)
		MR Egger	13	0.289	0.994 (0.983, 1.005)
	Osteoarthritis (hip)	Inverse variance weighted	14	0.006	0.900 (0.835, 0.971)
		Weighted median	14	0.003	0.859 (0.778, 0.949)
		MR Egger	14	0.121	0.843 (0.690, 1.030)
Phosphate to oleoyl-linoleoyl-glycerol (18:1 to 18:2) [2] ratio	Osteoarthritis	Inverse variance weighted	23	0.046	1.003 (1.000, 1.005)
		Weighted median	23	0.325	1.002 (0.998, 1.006)
		MR Egger	23	0.912	1.000 (0.994, 1.007)
	Osteoarthritis (hip)	Inverse variance weighted	23	0.025	1.073 (1.009, 1.141)
		Weighted median	23	0.012	1.116 (1.025, 1.216)
		MR Egger	23	0.732	0.975 (0.848, 1.122)
Tryptophan levels	Osteoarthritis	Inverse variance weighted	11	0.039	0.995 (0.991, 1.000)
		Weighted median	11	0.443	0.998 (0.992, 1.004)
		MR Egger	11	0.119	0.990 (0.978, 1.001)
	Osteoarthritis (hip)	Inverse variance weighted	11	0.013	1.119 (1.024, 1.223)
		Weighted median	11	0.019	1.156 (1.024, 1.306)
		MR Egger	11	0.654	1.059 (0.832, 1.348)
X-13431 levels	Osteoarthritis	Inverse variance weighted	18	0.043	1.002 (1.000, 1.003)
		Weighted median	18	0.01	1.003 (1.001, 1.005)
		MR Egger	18	0.075	1.003 (1.000, 1.006)
	Osteoarthritis (hip)	Inverse variance weighted	19	0.024	1.050 (1.007, 1.096)
		Weighted median	19	0.002	1.064 (1.022, 1.106)
		MR Egger	19	0.245	1.048 (0.971, 1.132)
X-21607 levels	Osteoarthritis	Inverse variance weighted	19	0.012	0.997 (0.994, 0.999)
		Weighted median	19	0.358	0.999 (0.995, 1.002)
		MR Egger	19	0.813	1.001 (0.996, 1.005)
	Osteoarthritis (hip)	Inverse variance weighted	20	0.026	0.928 (0.868, 0.991)
		Weighted median	20	0.075	0.942 (0.881, 1.006)
		MR Egger	20	0.231	1.076 (0.958, 1.208)
X-24757 levels	Osteoarthritis	Inverse variance weighted	12	0.022	1.004 (1.001, 1.008)
		Weighted median	12	0.147	1.004 (0.999, 1.009)
		MR Egger	12	0.905	1.001 (0.992, 1.010)
	Osteoarthritis (hip)	Inverse variance weighted	12	0.005	1.112 (1.032, 1.197)
		Weighted median	12	0.035	1.113 (1.007, 1.230)
		MR Egger	12	0.098	1.178 (0.988, 1.405)

Among the known plasma metabolites, two were found to be negatively associated with both Osteoarthritis and hip OA: Phenylacetylglutamate levels (OR[95%CI]: 0.995 [0.991, 0.999], 0.900 [0.835, 0.971]; respectively), and X-21607 levels (OR[95%CI]: 0.997 [0.994, 0.999], 0.928 [0.868, 0.991]; respectively). Conversely, three plasma metabolites showed a positive correlation with Osteoarthritis and hip OA: Phosphate to oleoyl-linoleoyl-glycerol (18:1 to 18:2) ratio (OR[95%CI]: 1.003 [1.000, 1.005], 1.073 [1.009, 1.141]; respectively), X-13431 levels (OR[95%CI]: 1.002 [1.000, 1.003], 1.050 [1.007, 1.096]; respectively), and X-24757 levels (OR[95%CI]: 1.004 [1.001, 1.008], 1.112 [1.032, 1.197]; respectively).

Tryptophan levels exhibited the most substantial positive correlation specifically with hip OA, as indicated by an odds ratio (OR) of 1.119 (95% confidence interval [CI]: 1.024, 1.223). Conversely, the association between tryptophan levels and general OA encompassing various joints including but not limited to the hip and knee was negative, with an OR of 0.995 (95% CI: 0.991, 1.000).

### 3.1 Casual association of plasma metabolites on osteoarthritis and knee osteoarthritis

**Figure 3:**
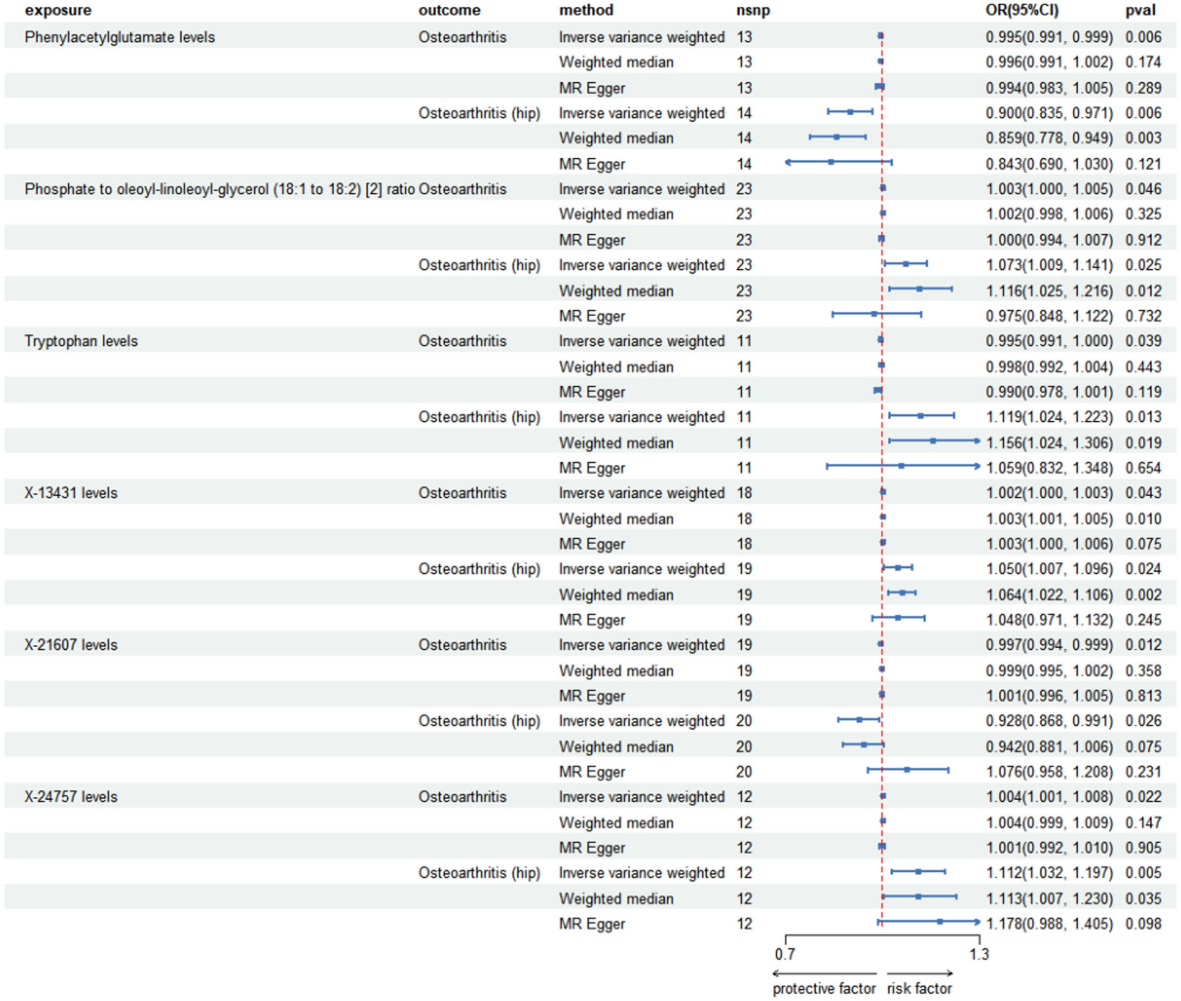
Forest plot for causal relationships of robust overlapped plasma metabolites on osteoarthritis and knee osteoarthritis utilizing Mendelian randomization. The change in the odds ratio (OR) of osteoarthritis, osteoarthritis (hip) per one-SD rise in the plasma metabolite level or metabolite ratio is shown by OR and 95% confidence interval.

After conducting a comprehensive series of quality control measures, we identified a significant correlation between 195 SNPs and Osteoarthritis. Furthermore, 203 SNPs were found to be associated specifically with knee Osteoarthritis (refer to Table 2; Supplementary Table S1, S3, for detailed information). We focused on including SNPs with *F* values exceeding 10 in our analysis. Utilizing the inverse variance weighted (IVW) method, we discovered that 13 plasma metabolites showed compelling evidence of association with both Osteoarthritis and hip OA (Figures 1, 3; Supplementary Tables S4, S6, S7, for comprehensive findings).

**Table 2:** Causal effects of significant plasma metabolites (*P_IVW_* < 0.05) on osteoarthritis and knee osteoarthritis.

Exposure	Outcome	Method	nsnp	*p* value	OR (95%CI)
(N(1) + N(8))-acetylspermidine levels	Osteoarthritis	Inverse variance weighted	22	0.03	1.003 (1.000, 1.006)
		Weighted median	22	0.01	1.005 (1.001, 1.008)
		MR Egger	22	0.182	1.004 (0.999, 1.008)
	Knee osteoarthritis	Inverse variance weighted	22	0.04	1.050 (1.002, 1.100)
		Weighted median	22	0.596	1.017 (0.955, 1.084)
		MR Egger	22	0.312	1.047 (0.960, 1.142)
Cysteinylglycine to glutamate ratio	Osteoarthritis	Inverse variance weighted	13	0.043	0.994 (0.988, 1.000)
		Weighted median	13	0.131	0.995 (0.989, 1.001)
		MR Egger	13	0.436	0.994 (0.979, 1.009)
	Knee osteoarthritis	Inverse variance weighted	14	0.004	0.894 (0.828, 0.965)
		Weighted median	14	0.013	0.886 (0.806, 0.975)
		MR Egger	14	0.179	0.883 (0.745, 1.047)
Ferulic acid 4-sulfate levels	Osteoarthritis	Inverse variance weighted	21	0.010	1.004 (1.001, 1.006)
		Weighted median	21	0.000	1.007 (1.003, 1.011)
		MR Egger	21	0.171	1.004 (0.999, 1.010)
	Knee osteoarthritis	Inverse variance weighted	21	0.044	1.041 (1.001, 1.083)
		Weighted median	21	0.004	1.081 (1.026, 1.140)
		MR Egger	21	0.121	1.066 (0.987, 1.152)
Glycine levels	Osteoarthritis	Inverse variance weighted	12	0.006	1.003 (1.001, 1.005)
		Weighted median	12	0.009	1.003 (1.001, 1.005)
		MR Egger	12	0.074	1.003 (1.000, 1.005)
	Knee osteoarthritis	Inverse variance weighted	13	0.029	1.042 (1.004, 1.080)
		Weighted median	13	0.022	1.039 (1.006, 1.074)
		MR Egger	13	0.228	1.035 (0.982, 1.090)
Glycine to serine ratio	Osteoarthritis	Inverse variance weighted	16	0.011	1.003 (1.001, 1.005)
		Weighted median	16	0.013	1.003 (1.001, 1.006)
		MR Egger	16	0.066	1.004 (1.000, 1.007)
	Knee osteoarthritis	Inverse variance weighted	16	0.001	1.077 (1.033, 1.123)
		Weighted median	16	0.016	1.052 (1.009, 1.095)
		MR Egger	16	0.397	1.029 (0.965, 1.097)
Histidine to alanine ratio	Osteoarthritis	Inverse variance weighted	13	0.005	0.994 (0.990, 0.998)
		Weighted median	13	0.198	0.996 (0.990, 1.002)
		MR Egger	13	0.456	0.996 (0.985, 1.007)
	Knee osteoarthritis	Inverse variance weighted	16	0.047	0.913 (0.835, 0.999)
		Weighted median	16	0.035	0.902 (0.820, 0.993)
		MR Egger	16	0.204	0.870 (0.708, 1.068)
N-acetylarginine levels	Osteoarthritis	Inverse variance weighted	16	0.025	1.004 (1.000, 1.007)
		Weighted median	16	0.282	1.003 (0.998, 1.007)
		MR Egger	16	0.151	1.005 (0.998, 1.012)
	Knee osteoarthritis	Inverse variance weighted	17	0.004	1.047 (1.015, 1.081)
		Weighted median	17	0	1.053 (1.023, 1.084)
		MR Egger	17	0.026	1.059 (1.012, 1.109)
N-palmitoyl-sphingosine (d18:1 to 16:0) to N-stearoyl-sphingosine (d18:1 to 18:0) ratio	Osteoarthritis	Inverse variance weighted	13	0.043	0.996 (0.992, 1.000)
		Weighted median	13	0.048	0.995 (0.990, 1.000)
		MR Egger	13	0.031	0.990 (0.981, 0.998)
	Knee osteoarthritis	Inverse variance weighted	13	0.002	0.907 (0.851, 0.966)
		Weighted median	13	0.179	0.951 (0.884, 1.023)
		MR Egger	13	0.917	1.007 (0.886, 1.145)
N-stearoyl-sphingosine (d18:1/18:0) levels	Osteoarthritis	Inverse variance weighted	13	0.019	1.006 (1.001, 1.011)
		Weighted median	13	0.012	1.007 (1.001, 1.012)
		MR Egger	13	0.142	1.014 (0.997, 1.033)
	Knee osteoarthritis	Inverse variance weighted	15	0.02	1.087 (1.013, 1.165)
		Weighted median	15	0.209	1.053 (0.972, 1.141)
		MR Egger	15	0.745	1.042 (0.817, 1.328)
Phosphate to phosphoethanolamine ratio	Osteoarthritis	Inverse variance weighted	6	0.009	0.992 (0.986, 0.998)
		Weighted median	6	0.02	0.991 (0.983, 0.999)
		MR Egger	6	0.522	0.995 (0.982, 1.008)
	Knee osteoarthritis	Inverse variance weighted	7	0.045	0.907 (0.824, 0.998)
		Weighted median	7	0.394	0.944 (0.826, 1.078)
		MR Egger	7	0.4	0.899 (0.716, 1.128)
X-17690 levels	Osteoarthritis	Inverse variance weighted	21	0.046	1.003 (1.000, 1.006)
		Weighted median	21	0.017	1.005 (1.001, 1.009)
		MR Egger	21	0.787	1.001 (0.994, 1.009)
	Knee osteoarthritis	Inverse variance weighted	21	0.018	1.079 (1.013, 1.149)
		Weighted median	21	0.234	1.044 (0.973, 1.121)
		MR Egger	21	0.649	1.037 (0.889, 1.210)
X-24757 levels	Osteoarthritis	Inverse variance weighted	12	0.022	1.004 (1.001, 1.008)
		Weighted median	12	0.147	1.004 (0.999, 1.009)
		MR Egger	12	0.905	1.001 (0.992, 1.010)
	Knee osteoarthritis	Inverse variance weighted	12	0.003	1.095 (1.032, 1.162)
		Weighted median	12	0.03	1.097 (1.009, 1.192)
		MR Egger	12	0.217	1.099 (0.955, 1.265)
Xanthurenate levels	Osteoarthritis	Inverse variance weighted	17	0.004	1.006 (1.002, 1.011)
		Weighted median	17	0.052	1.005 (1.000, 1.010)
		MR Egger	17	0.268	1.008 (0.995, 1.021)
	Knee osteoarthritis	Inverse variance weighted	16	0.049	1.063 (1.000, 1.129)
		Weighted median	16	0.108	1.065 (0.986, 1.149)
		MR Egger	16	0.484	1.071 (0.888, 1.291)

Among the known plasma metabolites, four plasma metabolites were negatively associated with osteoarthritis and knee osteoarthritis. These included the cysteinylglycine to glutamate ratio (OR [95% CI]: 0.994 [0.988, 1.000], 0.894 [0.828, 0.965], respectively), histidine to alanine ratio (OR [95% CI]: 0.994 [0.990, 0.998], 0.913 [0.835, 0.999], respectively), N-palmitoyl-sphingosine (d18:1 to 16:0) to N-stearoyl-sphingosine (d18:1 to 18:0) ratio (OR [95% CI]: 0.996 [0.992, 1.000], 0.907 [0.851, 0.966], respectively), and phosphate to phosphoethanolamine ratio (OR [95% CI]: 0.992 [0.986, 0.998], 0.907 [0.824, 0.998], respectively). On the other hand, nine plasma metabolites were positively associated with both osteoarthritis and knee osteoarthritis, including (N(1) + N(8))-acetylspermidine levels (OR [95% CI]: 1.003 [1.000, 1.006], 1.050 [1.002, 1.100], respectively), ferulic acid 4-sulfate levels (OR [95% CI]: 1.004 [1.001, 1.006], 1.041 [1.001, 1.083], respectively), glycine levels (OR [95% CI]: 1.003 [1.001, 1.005], 1.042 [1.004, 1.080], respectively), glycine to serine ratio (OR [95% CI]: 1.003 [1.001, 1.005], 1.077 [1.033, 1.123], respectively), N-acetylarginine levels (OR [95% CI]: 1.004 [1.000, 1.007], 1.047 [1.015, 1.081], respectively), N-stearoyl-sphingosine (d18:1/18:0) levels (OR [95% CI]: 1.006 [1.001, 1.011], 1.087 [1.013, 1.165], respectively), X-17690 levels (OR [95% CI]: 1.003 [1.000, 1.006], 1.079 [1.013, 1.149], respectively), X-24757 levels (OR [95% CI]: 1.004 [1.001, 1.008], 1.095 [1.032, 1.162], respectively), and xanthurenate levels (OR [95% CI]: 1.006 [1.002, 1.011], 1.063 [1.000, 1.129], respectively).

Among these associations, the strongest correlation was observed between X-24757 levels and knee osteoarthritis, with an OR of 1.095 (95% confidence interval: 1.032, 1.162).

### 3.2 Casual association of plasma metabolites on hip OA and knee osteoarthritis

**Figure 4:**
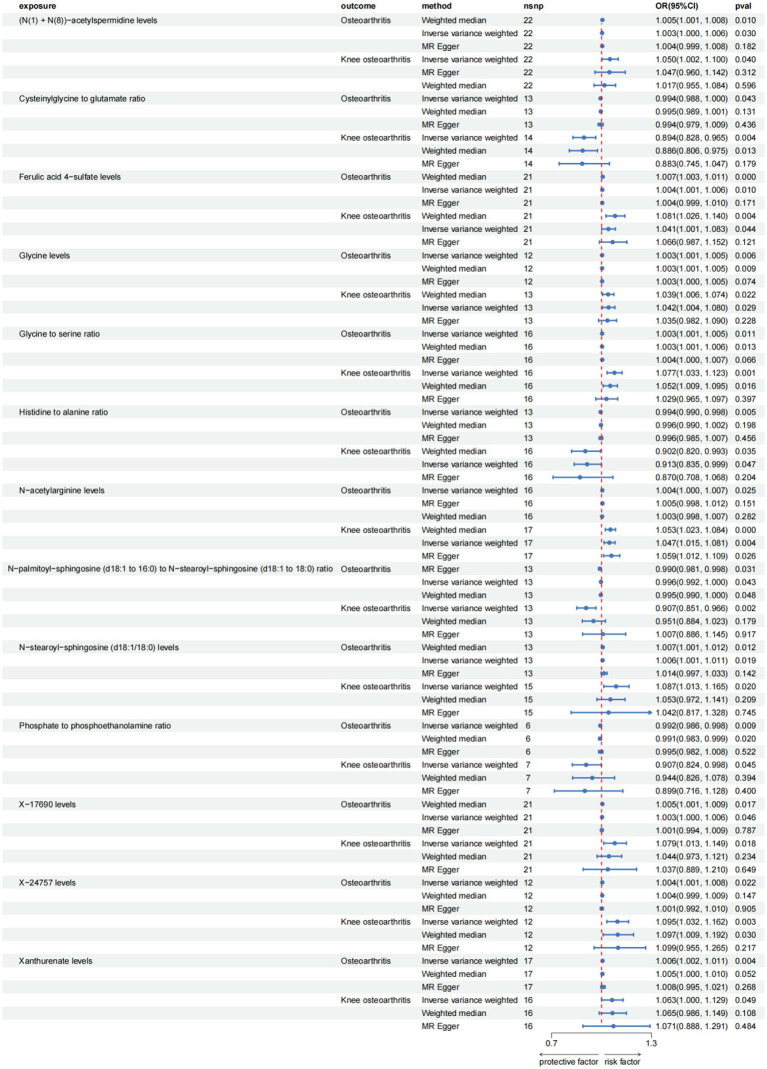
Forest plot for causal relationships of robust overlapped plasma metabolites on knee osteoarthritis and osteoarthritis (hip) utilizing Mendelian randomization. The change in the odds ratio (OR) of osteoarthritis, osteoarthritis (hip) per one-SD rise in the plasma metabolite level or metabolite ratio is shown by OR and 95% confidence interval.

After a comprehensive series of quality control procedures, we identified 104 SNPs that were significantly associated with hip OA and knee osteoarthritis, as detailed in Table 3; Supplementary Tables S2, S3. Specifically, we included SNPs with *F* values greater than 10. Employing the instrumental variable weighted (IVW) analysis, we detected a total of 9 plasma metabolites that exhibited strong evidence of association with both hip OA and knee osteoarthritis, as illustrated in Figures 1, 4; Supplementary Tables S5–S7.

**Table 3:** Causal effects of significant plasma metabolites (*P_IVW_* < 0.05) on osteoarthritis (hip) and knee osteoarthritis.

Exposure	Outcome	Method	nsnp	*p* value	OR (95%CI)
Ethylparaben sulfate levels	Osteoarthritis (hip)	Inverse variance weighted	12	0.024	1.118 (1.015, 1.231)
		MR Egger	12	0.072	1.232 (1.006, 1.509)
		Weighted median	12	0.224	1.075 (0.957, 1.209)
	Knee osteoarthritis	Inverse variance weighted	12	0.031	1.075 (1.007, 1.148)
		Weighted median	12	0.136	1.069 (0.979, 1.166)
		MR Egger	12	0.903	0.991 (0.862, 1.139)
Glucose-to-mannose ratio	Osteoarthritis (hip)	Weighted median	11	0.011	0.905 (0.838, 0.977)
		Inverse variance weighted	11	0.023	0.930 (0.873, 0.990)
		MR Egger	11	0.027	0.820 (0.708, 0.950)
	Knee osteoarthritis	Weighted median	11	0.007	0.918 (0.862, 0.977)
		Inverse variance weighted	11	0.043	0.943 (0.890, 0.998)
		MR Egger	11	0.241	0.915 (0.797, 1.051)
Indoleacetate levels	Osteoarthritis (hip)	Inverse variance weighted	22	0.04	1.075 (1.003, 1.152)
		Weighted median	22	0.173	1.067 (0.972, 1.171)
		MR Egger	22	0.493	1.058 (0.903, 1.238)
	Knee osteoarthritis	Inverse variance weighted	22	0.038	1.057 (1.003, 1.113)
		Weighted median	22	0.087	1.062 (0.991, 1.138)
		MR Egger	22	0.166	1.089 (0.970, 1.222)
Mannose to trans-4-hydroxyproline ratio	Osteoarthritis (hip)	Weighted median	10	0.02	1.152 (1.023, 1.297)
		Inverse variance weighted	10	0.044	1.098 (1.003, 1.202)
		MR Egger	10	0.047	1.308 (1.046, 1.636)
	Knee osteoarthritis	Weighted median	10	0.006	1.141 (1.039, 1.252)
		Inverse variance weighted	10	0.033	1.084 (1.007, 1.167)
		MR Egger	10	0.867	0.983 (0.811, 1.192)
Phenylalanine to tyrosine ratio	Osteoarthritis (hip)	Inverse variance weighted	10	0.028	0.917 (0.848, 0.991)
		Weighted median	10	0.037	0.896 (0.808, 0.993)
		MR Egger	10	0.568	0.952 (0.811, 1.119)
	Knee osteoarthritis	Weighted median	10	0.002	0.869 (0.795, 0.950)
		Inverse variance weighted	10	0.049	0.920 (0.847, 1.000)
		MR Egger	10	0.191	0.882 (0.743, 1.048)
Sphingosine to phosphate ratio	Osteoarthritis (hip)	Inverse variance weighted	9	0.02	1.115 (1.018, 1.222)
		Weighted median	9	0.114	1.102 (0.977, 1.243)
		MR Egger	9	0.265	1.217 (0.886, 1.671)
	Knee osteoarthritis	Inverse variance weighted	9	0.027	1.090 (1.010, 1.176)
		Weighted median	9	0.125	1.089 (0.977, 1.215)
		MR Egger	9	0.367	0.883 (0.686, 1.137)
Stearoylcholine levels	Osteoarthritis (hip)	Inverse variance weighted	10	0.006	0.879 (0.802, 0.963)
		Weighted median	10	0.016	0.871 (0.778, 0.975)
		MR Egger	10	0.624	0.944 (0.758, 1.177)
	Knee osteoarthritis	Inverse variance weighted	10	0.032	0.916 (0.845, 0.993)
		Weighted median	10	0.087	0.912 (0.822, 1.013)
		MR Egger	10	0.902	1.012 (0.838, 1.224)
X-12680 levels	Osteoarthritis (hip)	Inverse variance weighted	8	0.025	0.887 (0.799, 0.985)
		Weighted median	8	0.133	0.901 (0.787, 1.032)
		MR Egger	8	0.958	1.011 (0.698, 1.463)
	Knee osteoarthritis	Inverse variance weighted	8	0.043	0.918 (0.844, 0.997)
		Weighted median	8	0.205	0.934 (0.841, 1.038)
		MR Egger	8	0.79	1.043 (0.776, 1.401)
X-24757 levels	Osteoarthritis (hip)	Inverse variance weighted	12	0.005	1.112 (1.032, 1.197)
		Weighted median	12	0.035	1.113 (1.007, 1.230)
		MR Egger	12	0.098	1.178 (0.988, 1.405)
	Knee osteoarthritis	Inverse variance weighted	12	0.003	1.095 (1.032, 1.162)
		Weighted median	12	0.03	1.097 (1.009, 1.192)
		MR Egger	12	0.217	1.099 (0.955, 1.265)

Among the known plasma metabolites, four metabolites were negatively associated with both hip osteoarthritis (OA) and knee osteoarthritis. These included the glucose-to-mannose ratio (OR[95%CI]: 0.930 [0.873, 0.990], 0.943 [0.890, 0.998], respectively), the phenylalanine to tyrosine ratio (OR[95%CI]: 0.917 [0.848, 0.991], 0.920 [0.847, 1.000], respectively), stearoylcholine levels (OR[95%CI]: 0.879 [0.802, 0.963], 0.916 [0.845, 0.993], respectively), and X-12680 levels (OR[95%CI]: 0.887 [0.799, 0.985], 0.918 [0.844, 0.997], respectively). Additionally, five plasma metabolites were positively associated with both hip OA and knee osteoarthritis. These included ethylparaben sulfate levels (OR[95%CI]: 1.118 [1.015, 1.231], 1.075 [1.007, 1.148], respectively), indoleacetate levels (OR[95%CI]: 1.075 [1.003, 1.152], 1.057 [1.003, 1.113], respectively), the mannose to trans-4-hydroxyproline ratio (OR[95%CI]: 1.098 [1.003, 1.202], 1.084 [1.007, 1.167], respectively), the sphingosine to phosphate ratio (OR[95%CI]: 1.115 [1.018, 1.222], 1.090 [1.010, 1.176], respectively), and X-24757 levels (OR[95%CI]: 1.112 [1.032, 1.197], 1.095 [1.032, 1.162], respectively).

The study found that there was a significant positive association between ethylparaben sulfate levels and hip OA (OR[95%CI] = 1.118, 95% confidence interval [1.015, 1.231]).

### 3.3 Robust overlapped causal metabolites associated with osteoarthritis, hip OA, and knee osteoarthritis

**Figure 5:**
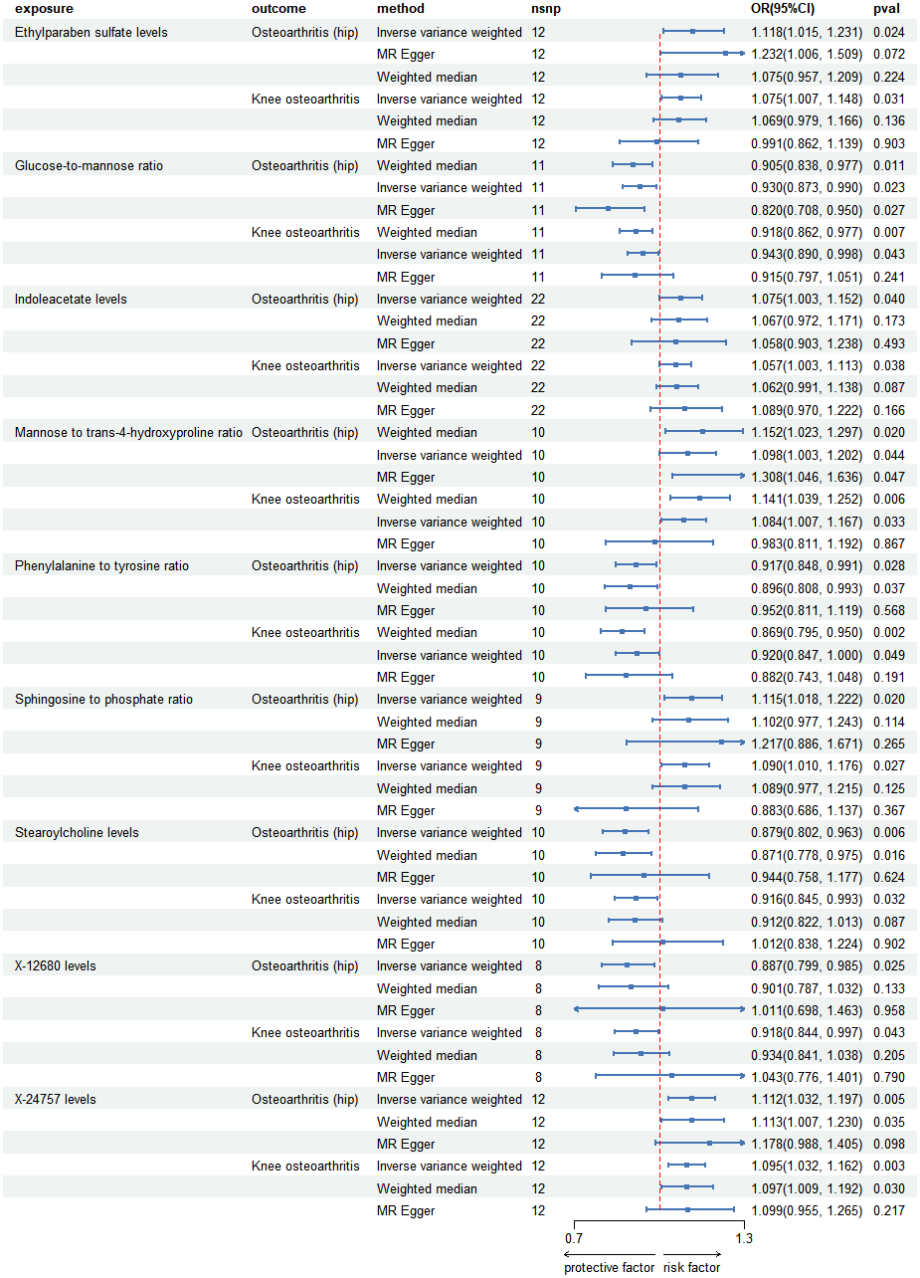
Venn diagram: identification of robust overlapped causal metabolites associated with osteoarthritis, osteoarthritis (hip) and knee osteoarthritis using Venn diagram. **(A)** Represents metabolites associated with osteoarthritis; **(B)** represents metabolites associated with osteoarthritis (hip); **(C)** represents metabolites associated with knee osteoarthritis. X-24757 is an unknown metabolite associated with osteoarthritis, osteoarthritis (hip) and knee osteoarthritis.

By conducting an analysis of the random effects between plasma metabolites and osteoarthritis, specifically focusing on hip OA and knee osteoarthritis, we have identified a plasma metabolite, X-2475 (Figure 5), that demonstrates consistent and robust random effects across all three types of osteoarthritis. It should be noted that X-24757 is currently an unidentified metabolite. In addition, our quality control measures have revealed that 12 SNPs are associated with osteoarthritis, hip OA, and knee osteoarthritis. Furthermore, our findings indicate a positive causal effect of X-24757 in all three types of osteoarthritis, with the strongest positive effect observed in hip OA [OR[95%CI]: 1.112 (1.032, 1.197)], followed by knee osteoarthritis [OR[95%CI]: 1.095 (1.032, 1.162)] and osteoarthritis [OR[95%CI]: 1.004 (1.001, 1.008)].

### 3.4 Sensitivity analysis

A series of sensitivity analyses were conducted to evaluate the robustness of the aforementioned findings. The presence of heterogeneity among plasma metabolites associated with the three types of osteoarthritis was assessed using Cochran’s Q test for the IVW method and MR Egger method, revealing no statistically significant heterogeneity (*p* > 0.05). Horizontal pleiotropy, a potential confounding factor, was further examined for all associations using MR-Egger’s intercept term and MR-PRESSO’s global test. The results indicated no significant evidence of horizontal pleiotropy among the plasma metabolites that were found to be significantly associated with the three types of osteoarthritis. Moreover, the direction of effect obtained from all three methods, including the IVW method, was consistent. The IVW radial MR Results demonstrated that the corrected findings were in concordance with the pre-corrected results (Supplementary Tables S8–S10).

Plasma metabolites X-24757, known for their strong random effects in all three types of osteoarthritis, were carefully examined in this study using various analytical techniques to ensure data reliability. Sensitivity analysis, scatter plots (Figure 5), and funnel plots (Figure 2) were utilized to identify and exclude any potential outliers and assess the presence of horizontal pleiotropy. The results obtained indicated no significant heterogeneity or horizontal pleiotropy (Supplementary Figures S3–S5). To further validate the findings, a leave-one-out (LOO) test was conducted, whereby each single nucleotide polymorphism (SNP) was eliminated iteratively to assess the impact on the overall effect estimate. Remarkably, the effect estimate remained consistent with the total Mendelian randomization results, demonstrating the robustness of the findings (Supplementary Figure S6). Additionally, Steige’s directional test was performed, and the analysis results were found to align with the expected direction (Supplementary Table S11).

### 3.5 Metabolic pathway analysis

Metabolic pathway analysis revealed the presence of six significant metabolic pathways in the hip OA phenotype, with only four pathways showing statistical significance in relation to pathogenesis (Supplementary Tables S11, S12). The Amino Sugar Metabolism (*p* = 0.089), Fatty Acid Biosynthesis (*p* = 0.095), and Aspartate Metabolism (*p* = 0.097) pathways were found to be associated with the development of osteoarthritis in the hip joint. None of the pathways investigated were found to be associated with osteoarthritis in general or knee osteoarthritis specifically. Notably, acetylarginine was identified as a common mediator in all of the metabolic pathways identified.

## 4 Discussion

This study investigated the potential causal effects of 1,091 circulating metabolites and 309 metabolite ratios on the development of three OA phenotypes using Mendelian randomization analysis. The research offers new insights into comprehending the intricate metabolic mechanisms of OA. By integrating two large-scale GWAS datasets and employing a robust MR Design, the analysis identified several metabolites associated with the genetic risk for OA. The study pinpointed 94 plasma metabolites linked to OA, 60 to hip osteoarthritis, and 106 to knee osteoarthritis. Notably, through random effects analysis on plasma metabolites and osteoarthritis subtypes, namely hip OA and knee OA, the study revealed substantial consistent effects of X-2475 across all three types of OA. Furthermore, four crucial acetylarginine-mediated metabolic pathways were also identified in hip OA. These results offer further understanding of the causal mechanisms underlying OA, unveil the impact of gene–environment interactions on OA development, and offer potential insights for advancing precision medicine.

In the past decade, OA has gained increasing recognition as a disease related to metabolism. This is due to its association with various metabolic disorders, as well as the continuous discovery of metabolites and metabolic pathways associated with OA through metabolomics studies (5, 6, 18, 19). Typical sample sources for metabolomic identification include blood, synovial fluid, cartilage, and subchondral bone (20). Among these, blood is considered an excellent source due to its abundance of detectable metabolites and ease of obtaining large sample sizes, facilitating the identification of circulating markers for OA risk assessment (21, 22). Plasma metabolomics studies have revealed altered metabolic characteristics in OA patients, with the most commonly observed metabolites being amino acids, taurine, and phospholipids (18, 19). Our study not only confirmed the presence of OA-specific metabolic profiles, but also identified key plasma metabolites and metabolic pathways that contribute to the pathogenesis of OA.

Our study has identified specific metabolic pathways that are associated with the pathogenesis of hip OA. These include Ethanol Degradation, Amino Sugar Metabolism, Fatty Acid Biosynthesis, and Aspartate Metabolism. The significance of these findings underscores their potential role in the development of hip OA. The importance of the ethanol degradation pathway may indicate a potential link between alcohol consumption and OA. This is consistent with a previous study by He K, Huang H et al. which suggests that alcohol consumption may interact with C-reactive protein and self-reported OA (23). On the other hand, the association of amino sugar metabolism may be related to the biochemical composition of cartilage (24). The significance of fatty acid synthesis may indicate a link between abnormal fat metabolism and the pathogenesis of OA, while the association of a spartate metabolism may involve the process of amino acid metabolism in the articular cartilage.

Notably, all of these metabolic pathways were associated with acetylarginine, an acetylated derivative of arginine, suggesting a common molecular mediating mechanism. Arginine is a semi-essential amino acid involved in the urea cycle and arginine/proline metabolism (25). In the human body, arginine is metabolized to urea and ornithine through the arginase pathway, while nitric oxide (NO) and citrulline are produced by the nitric oxide synthase (NOS) pathway (25). The anti-inflammatory and antioxidant effects of arginine have been demonstrated in various tissues and cells, mainly through nitric oxide (26). Previous studies have shown that NO and its REDOX derivatives may play a protective role in joints by inhibiting inflammatory pathways and reducing immune cell infiltration (26, 27). Metabolomic analysis indicates arginine depletion in patients with OA and suggests that this may be due to hyperactive arginine catabolism (28). Arginine deficiency has been identified as one of the three clinical endotypes of OA, along with muscle weakness and low inflammatory OA (29). Multi-omics analysis of injury-induced OA animal models has also observed enrichment of arginine metabolism-related genes, highlighting the regulatory role of the arginine metabolic pathway in OA (9). Specifically, Choi et al. found that the gene encoding Arg-II, an arginine-metabolizing enzyme (28), was upregulated in cartilage samples from human OA patients and mouse models. They also demonstrated that adenovirus-mediated overexpression of Arg-II in mouse joint tissues led to OA pathogenesis, while genetic elimination of Arg2 (Arg2 −/−) in mice abolished pathological manifestations of OA. In addition, Li et al. found that exogenous arginine supplementation helped reduce lipid peroxidation and inflammatory responses in osteoblast-osteoarthritis cells (30), providing a new therapeutic target for OA treatment. Our results further support these findings and highlight the importance of arginine as a protective factor in the progression of hip OA.

Previous studies have demonstrated that dysregulation of lipid metabolism is a prominent pathophysiological feature of OA (31, 32). Phospholipids, essential constituents of synovial fluid responsible for joint lubrication, have been found to exhibit disorders in phosphatidylcholine/lysophosphatidylcholine metabolism in OA, according to increasing experimental evidence (32–34). These metabolic changes hold promise as potential circulating markers and drug targets for OA (32–34). Moreover, the shared metabolic pathway of phosphatidylcholine/lysophosphatidylcholine metabolism has been identified between OA and metabolic diseases, such as diabetes, which may explain the concurrent occurrence of these conditions (18, 35). Although the involvement of oleoyl glycerolphosphocholine in OA remains unexplored, previous studies have established its potential as a biomarker for insulin resistance and impaired glucose metabolism (36).

Our findings provide additional genetic evidence in humans linking specific metabolic disorders to the development of OA. For instance, we observed that elevated levels of (N1 + N8)-acetylspermidine, Ferulic acid 4-sulfate, Glycine, N-acetylarginine, N-stearoyl-sphingosine (d18:1/18:0), X-17690, and Xanthurenate are common risk factors for OA and knee OA. Among these, (N1 + N8)-acetylspermidine may be involved in cell proliferation and apoptosis processes, which could contribute to OA pathogenesis. Ferulic acid 4-sulfate may exert antioxidant and anti-inflammatory effects, which may be relevant to OA development. Glycine, being an amino acid, may have implications for cartilage structure and function. N-acetylarginine could be related to nitric oxide metabolism and inflammatory responses. N-stearoyl-sphingosine, a sphingolipid metabolite, might play a role in cell signaling and apoptosis. Xanthurenate may be associated with oxidative stress and inflammation. However, the mechanisms underlying the influence of other metabolites on OA are not fully elucidated and require further experimental investigation. Moreover, we observed that several metabolites display protective effects against OA phenotypes. Specifically, increased levels of Phenylacetylglutamate, X-21607, Stearoylcholine, and X-12680 were associated with reduced risk of OA or its phenotypes. Nevertheless, the mechanisms mediating the effects of these metabolites on OA remain incompletely understood and necessitate further experimental exploration.

The discovery of metabolite ratios provides valuable insights into the understanding of metabolic crosstalk in OA. Specifically, the ratio of Phosphate to oleoyl-linoleoyl-glycerol (18:1 to 18:2) (2) is positively associated with OA and hip OA, indicating that the imbalance between these metabolites may influence the pathogenesis of OA and hip arthritis. Conversely, the ratios of N-palmitoyl-sphingosine (d18:1 to 16:0) to N-stearoyl-sphingosine (d18:1 to 18:0), Phosphate to phosphoethanolamine, Histidine to alanine, and Cysteinylglycine to glutamate are negatively correlated with OA and knee OA, suggesting that the imbalance of these amino acids and phospholipids may also impact the development of OA and knee OA. Furthermore, the ratios of Mannose to trans-4-hydroxyproline and Sphingosine to phosphate are positively correlated with both hip OA and knee osteoarthritis. Overall, the modulation of the interdependence and network of metabolites through integrated therapies represents a promising frontier in the study of OA metabolism.

Metabolic pathways play a causal role in the development of hip osteoarthritis, and their involvement in the pathogenesis of osteoarthritis has been extensively studied. Specifically, pathways such as arginine biosynthesis, arginine and proline metabolism, and lipid metabolism have been identified as being implicated in the pathogenesis of osteoarthritis (28, 30). In addition, our research has demonstrated an association between plasma metabolites and both hip and knee osteoarthritis. Through a random effects analysis, we observed a strong and consistent positive causal effect of plasma metabolite X-2475 levels across all three types of osteoarthritis. This finding suggests the presence of a plasma metabolite and metabolic pathway that are strongly linked to the risk of all types of osteoarthritis. Further experimental studies are warranted to validate the causal effect of X-2475 levels.

The association between plasma metabolites and both hip and knee osteoarthritis means that inclusion of tryptophan and X-24757 in early OA Screening helps early identification of people at risk. When tryptophan or X-24757 levels are elevated, the individual is at potential risk of developing OA. The individual should be included in the risk group, diagnosed with disease progression and targeted for early intervention.

Our study possesses several notable strengths. Firstly, a key strength of this study lies in its extensive coverage of genetic variables on a large scale, enabling us to analyze the relationship between blood metabolites and various OA phenotypes. Specifically, a total of 1,400 metabolites were included in our analysis, enhancing the comprehensiveness of our investigation. Secondly, we sought to mitigate the impact of reverse causality and residual confounding through the utilization of a MR design. This approach effectively minimized the potential influence of variable polymorphisms, thereby bolstering the robustness of the study’s findings pertaining to the causal relationship between metabolites and OA risk. The inclusion of rigorous sensitivity analyses further strengthens the reliability of our conclusions.

Our study has several limitations that need to be acknowledged. Firstly, the GWAS database used in this study only includes data from European populations, raising questions about the generalizability of our findings to non-European populations. Secondly, relying on patient self-reported information introduces the potential for misdiagnosis in cases of OA. Thirdly, while MR methods were used to confirm the association between plasma metabolites and OA risk, they can only offer preliminary evidence, leaving the underlying biological mechanisms unclear. Further comprehensive investigations are necessary to fully understand this relationship. Fourthly, the presence of unidentified metabolites poses a challenge for further research on their association with disease. Fifthly, the limited number of SNPs meeting the standard bioinformatic threshold of *p* < 5 × 10^−8^ may complicate matching IVs in the outcome and weaken associations. We opted for a less stringent significance level of 5 × 10^−5^ to select SNPs, but this choice comes with the risk of weak instrumental variable bias, requiring caution in interpreting our results. Lastly, discrepancies between causal outcomes from analytical techniques like MR-Egger and the primary IVW method suggest potential pleiotropy. While sensitivity analyses did not reveal obvious horizontal pleiotropy, testing the hypothesis that genetic instruments only affect outcomes through exposure factors without vertical pleiotropy remains challenging due to incomplete knowledge of the biological effects of these SNPs. Last, the current understanding of the relationship between plasma metabolites and OA is limited, potentially leading to inaccuracies. It is crucial to acknowledge the diverse spectrum of OA severity. Further clinical data on age and sex demographics are advised.

## 5 Conclusion

This study presents a systematic MR Analysis using a large-scale two-sample GWAS to examine the causal relationship between serum metabolites and various OA phenotypes. The findings provide initial evidence for the influence of metabolic disorders on the risk of OA. The results indicate that Tryptophan levels are strongly associated with the risk of hip OA, while X-24757 levels are most associated with the risk of knee Osteoarthritis. Additionally, Ethylparaben sulfate levels are found to be closely associated with the risk of hip OA. Notably, an unidentified plasma metabolite, X-2475, demonstrates a robust positive causal effect across all types of osteoarthritis, highlighting its significance as a plasma metabolite consistently linked to OA risk. Furthermore, the study identifies four significant metabolic pathways, all involving acetylarginine, in the context of hip osteoarthritis. These findings offer novel insights into OA biomarkers and pathways, which can contribute to prevention strategies and clinical interventions. Moreover, this research identifies promising circulating metabolic biomarkers for OA screening and prevention, as well as potential candidate molecules for further mechanistic investigation and drug target selection.

## Data availability statement

The datasets presented in this study can be found in online repositories. The names of the repository/repositories and accession number(s) can be found in the article/Supplementary material.

## Ethics statement

The studies involving humans were approved by the Ningbo Second Hospital, No. 41, Northwest Street, Haishu District, Ningbo, China. The studies were conducted in accordance with the local legislation and institutional requirements. The participants provided their written informed consent to participate in this study.

## Author contributions

QF: Conceptualization, Writing – review & editing. XY: Conceptualization, Writing – review & editing. WW: Data curation, Writing – review & editing. XH: Formal analysis, Writing – review & editing. JZ: Visualization, Writing – review & editing. JW: Writing – original draft, Writing – review & editing. YW: Writing – original draft, Writing – review & editing.

## Footnotes

^1^ https://www.ebi.ac.uk/gwas/publications/36635386

^2^ https://gwas.mrcieu.ac.uk/

^3^ https://www.metaboanalyst.ca/
