# Supplementary material for: Causal association of genetically determined plasma metabolites with osteoarthritis: a two-sample Mendelian randomization study
Source: Front Med (Lausanne). 2024 Jun 28;11:1396746. doi: 10.3389/fmed.2024.1396746 (PMC11245738; doi:10.3389/fmed.2024.1396746)
Supplement: Supplementary file 2 [file Data_Sheet_2.pdf]

Supplementary Material

Supplementary Figures and Tables

Supplementary Figures

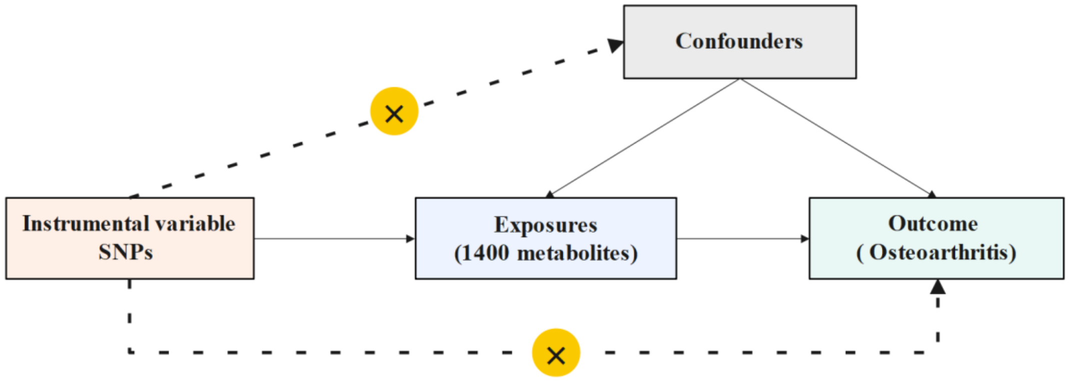

Supplementary Figure 1. Three key hypotheses of instrumental variables.

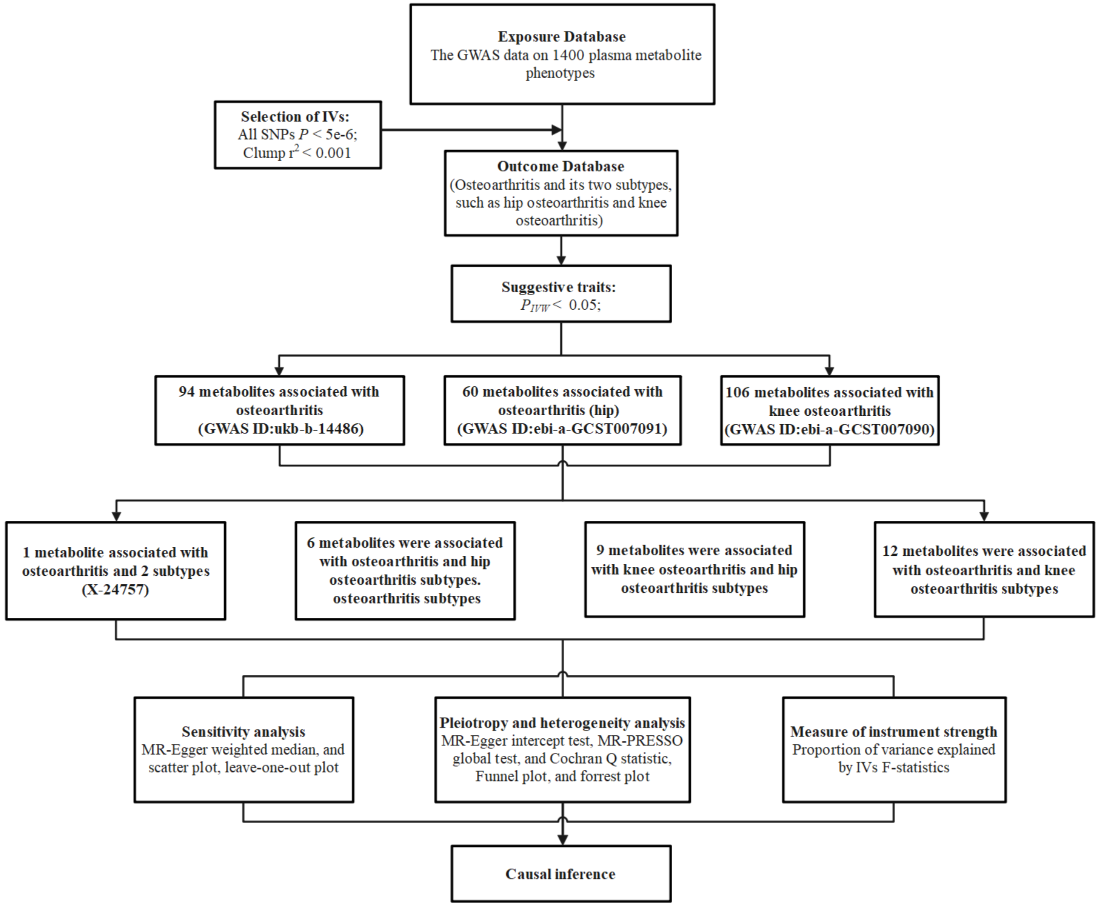

Supplementary Figure 2. Flow diagram.

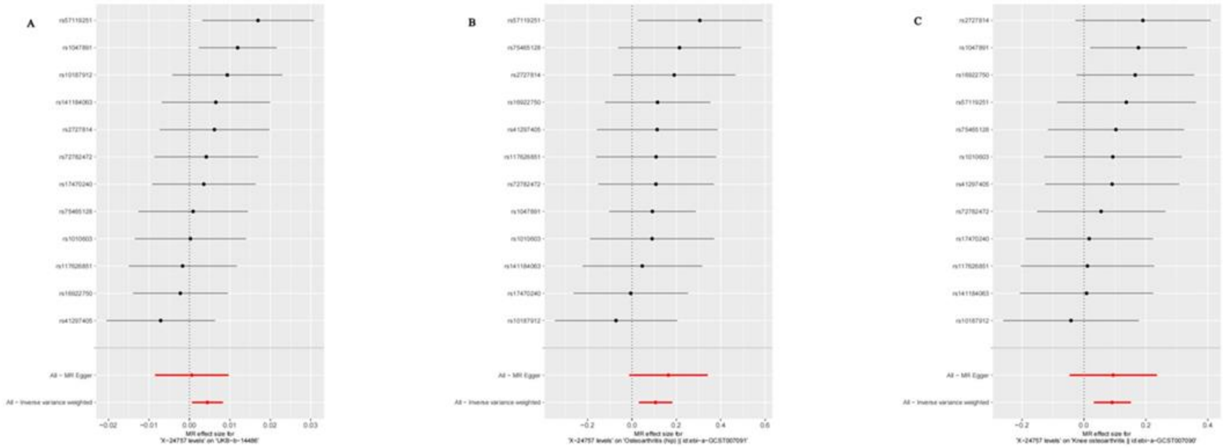

Supplementary Figure 3. Forest Plots depicting the overlapping metabolites (X-24757) associated with osteoarthritis, osteoarthritis (hip) and knee osteoarthritis using Mendelian Randomization Analysis.

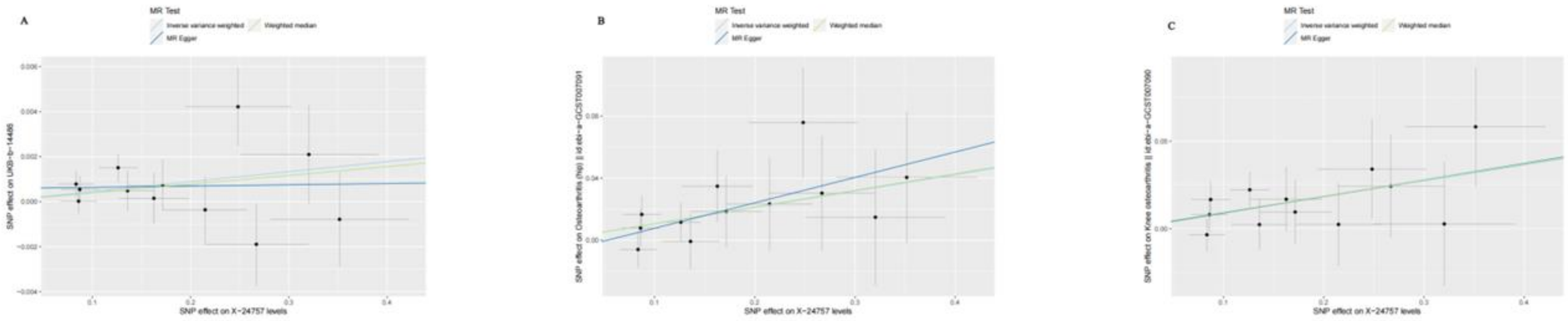

Supplementary Figure 4. Scatter Plots depicting the overlapping metabolites (X-24757) associated with osteoarthritis, osteoarthritis (hip) and knee osteoarthritis using Mendelian Randomization Analysis.

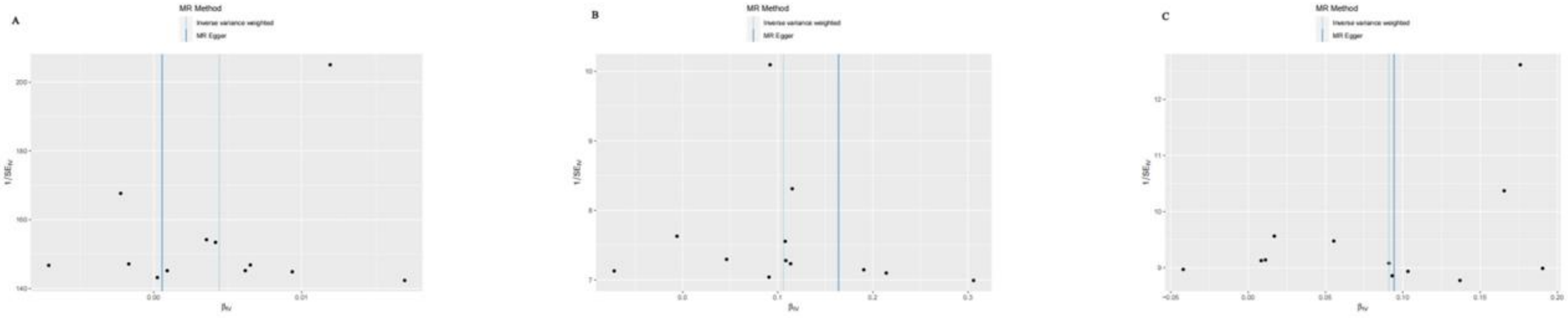

Supplementary Figure 5. unnel Plots depicting the overlapping metabolites (X-24757) associated with osteoarthritis, osteoarthritis (hip) and knee osteoarthritis using Mendelian Randomization Analysis.

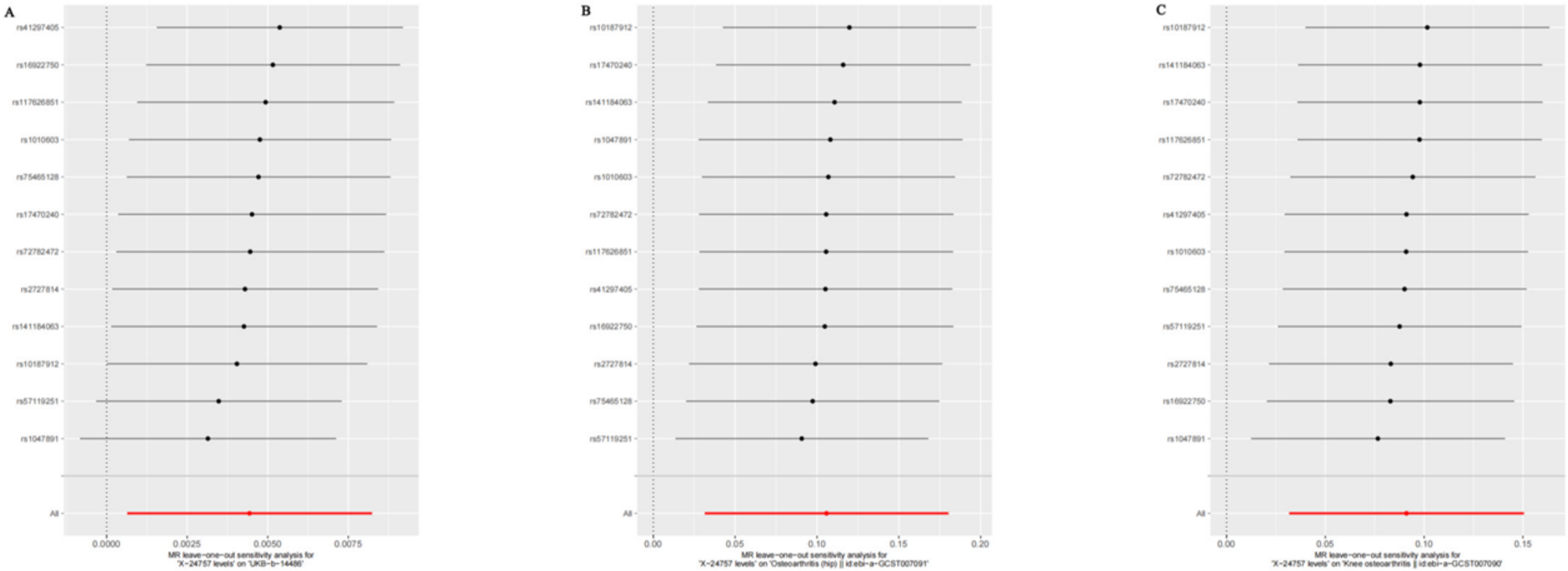

Supplementary Figure 6. Leave-One-Out Plots depicting the overlapping metabolites (X-24757) associated with osteoarthritis, osteoarthritis (hip) and knee osteoarthritis using Mendelian Randomization Analysis.
